# Army Medical Corps

**Published:** 1919-04

**Authors:** 


### ARMY MEDICAL CORPS

Representative Dyer proposes in H. R. 13344 to amend the national defense act, as follows:

That the medical department of the Army shall consist of one surgeon general with the rank of major general, who shall be chief of said department; assistant surgeon generals in the ratio of one-half of one per centum of the total number of officers of the medical department provided by law, the assistant surgeon generals to be equally distributed in the grades of major general and brigadier general of the medical corps, the dental corps, and the veterinary corps authorized by law, the commissioned officers of which shall be citizens of the United States, the enlisted force of the medical department of the Army as now provided by law.

Sec. 2. That commissioned officers of the medical corps below the rank of brigadier general shall be proportionately distributed in the several grades as now provided by law for the medical corps of the Navy.

Sec. 3. That hereafter the President shall be authorized to fill any vacancy that may occur in the commissioned personnel of the permanent establishment of the medical department of the Army of the United States by selection from the medical officers of the United States Army of not less than one year's continuous active service; Provided, That the selection shall be upon the efficiency rating and satisfactory examination of the officer, and where two or more officers are of equal rating selection will favor the one of the longest service in the United States Army, Volunteer Army, National Guard, officers' reserve corps or National Army: Provided further, That the officers so selected will be commissioned without loss of rank in the permanent establishment of the medical department of the Army of the United States, and such officers so commissioned shall be entitled to all pay, promotion, and allowances of officers of like rank in the permanent establishment of the Army of the United States, excepting that the rate of retirement pay, shall be one-thirteenth of the present retirement pay, as now prescribed by law, for each year's service as enlisted man; or commissioned officer, or the total of such services in the United States Army, Volunteer Army, National Guard, officers' reserve corps, National Army, dental corps, dental reserve, or enlisted personnel in the Army or National Guard in any of the various departments of the Army of the United States: And provided further, That at the age now prescribed by law for retirement an officer to receive retirement pay shall have not less than 15 years' service in any or all branches enumerated in this act: And provided further, That the provisions of this act shall in like manner govern the filling hereafter of all vacancies in the dental corps of the United States Army: And provided further, That the ratio of dental surgeons now and hereafter commissioned shall be two dental surgeons per thousand of enlisted strength of the Army of the United States: And provided further, That all laws or parts of laws, insofar as they are inconsistent with the provisions of this act, are hereby repealed.—*Army and Navy Register.*

